# A Rare Case of Methicillin-Resistant Staphylococcus aureus (MRSA) Enterocolitis Treated With Oral Vancomycin

**DOI:** 10.7759/cureus.20143

**Published:** 2021-12-03

**Authors:** Ariana R Tagliaferri, Mohamed Elagami, Gabriel Melki, Yasmeen Sultana, Ashesha Mechineni

**Affiliations:** 1 Internal Medicine, St. Joseph's Regional Medical Center, Paterson, USA; 2 Medicine, St. Joseph's Regional Medical Center, Paterson, USA; 3 Infectious Disease, St. Joseph's Regional Medical Center, Paterson, USA

**Keywords:** vancomycin, methicillin-resistant staphylococcus aureus, enterocolitis, diarrhea, clostridium infections

## Abstract

Historically, methicillin-resistant Staphylococcus aureus (MRSA) was thought to be the primary pathogen in pseudomembranous enterocolitis associated with antibiotic use or recent abdominal surgery; however, *Clostridioides difficile *was later identified as another more common pathogen. Since the eclipse of *C. difficile* the workup of hospital-acquired diarrhea now utilizes nucleic acid amplification rather than stool cultures and longer includes the investigation of other less common pathogens. Consequently, the diagnosis of MRSA enterocolitis has faded. It is imperative to consider more sinister pathogens not routinely covered in laboratory testing as MRSA enterocolitis infections have been known to progress to severe systemic infections and thus the delay or misdiagnosis can result in inappropriate treatment, prolonged hospitalizations, sepsis and/or death. Herein we present a case of a patient who presented with laboratory diagnosed MRSA enterocolitis in the absence of recent abdominal surgery or antibiotic use and was successfully treated with oral vancomycin.

## Introduction

Nosocomial diarrhea can affect up to one-third of all hospitalized patients, especially in the immunocompromised or postsurgical demographic [1]. Tracing back to the 1950s, methicillin-resistant Staphylococcus aureus (MRSA) was thought to be the primary pathogen in pseudomembranous enterocolitis associated with antibiotic use or recent abdominal surgery [2-4]. In one study, 74% of MRSA colitis cases (581/782) were associated with antibiotics, followed by gastrointestinal surgeries (18% 140/782) and then patients with a history of inflammatory bowel disease (2% 12/782) [1]. Of those patients included in the follow-up, 66% died likely from the presence of systemic MRSA infection. Since then, various antibiotics have been associated with nosocomial diarrhea and in every case MRSA is present in the stool; however, fatality rates have declined over time due to expansion and accessibility to antibiotics [3,4].

By the late 1970s, *Clostridioides difficile* (*C. difficile*) was identified as another pathogen causing pseudomembranous colitis in the setting of antibiotic use, but in the absence of MRSA positive cultures [4]. Managing diarrhea in hospitalized patients can thus be quite complex and requires multiple investigations in order to treat appropriately, without causing further nosocomial infection [1]. Since the eclipse of *C. difficile *the workup of hospital-acquired diarrhea now utilizes nucleic acid amplification thus favoring the testing and treatment for *C. difficile*, without utilization of any stool cultures [1]. Consequently, the diagnosis of MRSA enterocolitis has become only a historical cause of enterocolitis [1]. However, it is imperative to consider historical, but more sinister pathogens not routinely covered in laboratory testing, as MRSA enterocolitis infections have been known to progress to severe systemic infections and thus the delay or misdiagnosis can result in inappropriate treatment, prolonged hospitalizations, sepsis and/or death [5]. The fatality and diagnosis of MRSA enterocolitis may have declined since the 1950s, but it is a distinct clinical entity that should be properly investigated and treated in hospitalized patients [6].

This manuscript was published as an abstract in the American Journal of Gastroenterology (Volume 116) and presented as a poster in the Annual American College of Gastroenterology Conference in Las Vegas in October 2021. 

## Case presentation

This is a case of a 71-year-old female who presented to the hospital complaining of diarrhea lasting seven days. Her past medical history is significant for recurrent abdominal wall hernias that was treated surgically with mesh wall repairs, hyperlipidemia, coronary artery disease, arthritis, congestive heart failure with preserved ejection fraction, anemia of chronic disease, lumbar spinal stenosis, hypertension and morbid obesity. The patient described the diarrhea as non-bloody, brown, and soft watery stool. The patient normally has at least one bowel movement per day. When the diarrhea started, the patient reported having more frequent bowel movements, with an average of three per day. The bowel movements were explosive in nature and with a large volume output. She also reported that defecation was associated with abdominal cramping in the patient’s lower abdomen. The diarrhea was associated with tenesmus, with incontinence on occasion. Pertinent review of systems was negative for fever, weight loss, headache, dizziness or near syncopal events, nausea, vomiting, hematemesis, chest pain, shortness of breath, numbness or tingling, melena or hematochezia. The patient also denied any recent changes in diet, recent consumption of food from restaurants, recent travel, or sick contacts. Two years prior to this hospitalization the patient underwent a colonoscopy for hematochezia which showed a solitary 5-millimeter ulcer with stigmata of recent bleeding, mild diverticulosis in the sigmoid and descending colon without evidence of bleeding. On presentation, the patient was hemodynamically stable and afebrile. A complete metabolic panel (CMP), complete blood count (CBC) and blood cultures were obtained (Tables 1, 2). The patient was found to have acute kidney injury (AKI), however serum electrolytes, white blood cell count, and hemoglobin were all within normal limits.

**Table 1:** Chemistry panel and lactic acid.

Lab Test	Result (units)	Lab Test	Result (units)
Sodium	136 mEq/L	Alkaline Phosphatase	81 unit/L
Potassium	4.8 mEq/L	Aspartate aminotransferase	24 unit/L
Chloride	103 mEq/L	Alanine aminotransferase	19 unit/L
Bicarbonate	27 mEq/L	Phosphorous	4.2 mg/dL
Glucose	88 mg/dL	Magnesium	2.1 mg/dL
Calcium	9.6 mg/dL	Lipase	20 unit/L
Blood urea nitrogen	46 mg/dL	Lactic Acid	0.9 mmol/L
Creatinine	1.56 mg/dL	Albumin	3.8 g/dL
Total Bilirubin	0.6 mg/dL	Total Protein	7.3 g/dL

**Table 2:** Complete blood count without differential.

Lab Test	Result (units)	Lab Test	Result (units)
White blood cell	5.7 x10^3^/mm^3^	Mean Corpuscular Hemoglobin	30.2 pg
Red blood cell	4.27 x10^6^/mm^3^	Mean Corpuscular Hemoglobin Concentration	32.8 g/dL
Hemoglobin	12.9 g/dL	Red Blood Cell Distribution Width	15%
Hematocrit	39.3%	Platelets	254 K/mm^3^
Mean Corpuscular Volume	92.0 fL	Mean Platelet Volume	10.3 fL

The patient underwent computed tomography (CT) scan of abdomen and pelvis without contrast, which revealed a large anterior abdominal wall hernia containing small bowel loops without obstruction, and signs of colitis. Stool *C. difficile* toxin and PCR were negative. She was admitted for gastroenteritis and treated with intravenous hydration. As her diarrhea persisted, stool cultures were sent on hospital day 4, which grew MRSA, and the MRSA PCR nares were positive. Thus, the diagnosis of MRSA colitis was established. By hospital day 7, the diarrhea significantly improved with oral vancomycin (125 mg every six hours) and intravenous hydration, however her AKI persisted. She ultimately required 12 days of hospitalization and was discharged home with instructions to continue the same dose of vancomycin for a total of 14 days. On follow-up, the patient’s diarrhea had completely resolved.

## Discussion

In the mid-twentieth century, *Staphylococcus aureus* (*S. aureus*) was considered one of the major causes of antibiotic-associated pseudomembranous colitis [7]. After the identification of *Clostridioides difficile* (*C. difficile*) as a cause of antibiotic-associated pseudomembranous colitis (in 1978), the appreciation of *S. aureus* as a potential etiology has declined and been disputed. Subsequently, routine stool cultures in the hospital settings were discontinued in the United States and physicians no longer seek out S. aureus as a cause of pseudomembranous colitis [8]. *C. difficile* is now identified as the causative agent in approximately 30% of antibiotic-associated diarrhea and as the primary cause of antibiotic-associated colitis [9].

Published data show that risk factors MRSA colitis include recent antibiotic use, acid -suppressive therapy and recent abdominal surgery. Typically, MRSA colitis presents with fever, abdominal distention, and watery diarrhea, which often leads to severe dehydration, shock, and even multi-organ failure. Stool gram stains and cultures are the mainstay of diagnosis, while imaging studies and colonoscopy may aid in confirming the diagnosis or ruling out other disorders [10]. Although our patient demonstrated colitis on the CT scan, a repeat colonoscopy may have been useful in further confirming the cause of her diarrhea. Treatment with oral vancomycin has been shown to be effective, however, the advanced disease may require surgery [11].

## Conclusions

In this case report, we present a case of MRSA colitis that presented in the absence of recent antibiotic use or abdominal surgery. This case report serves to increase awareness of MRSA colitis as a distinct clinical entity from C. difficile, including the pathogenesis, gross and microscopic pathology, investigations, and management. In the absence of *C. difficile* toxin or PCR positivity, one should investigate for other pathogens of nosocomial diarrhea. Although the fatality rates have declined since 1950, MRSA colitis is still more sinister and one should treat accordingly if diagnosed. Early diagnosis requires high clinical suspicion and early treatment is of great importance to prevent its progress to severe systemic infections.
